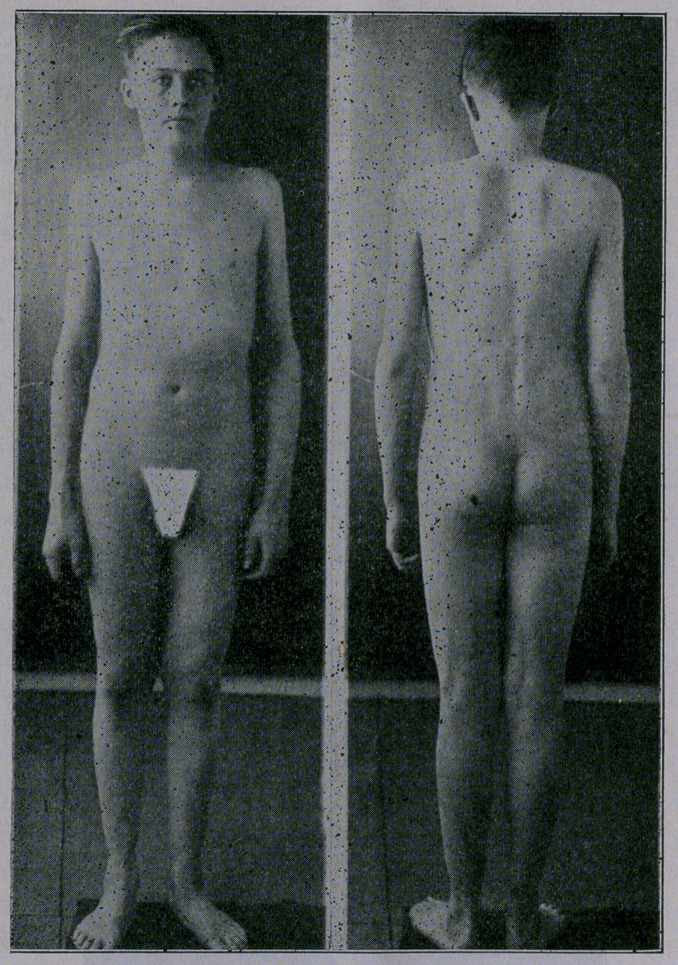# Mal-Nutrition and Its Treatment

**Published:** 1914-11

**Authors:** Emile Brunor

**Affiliations:** New York


### Mal-Nutrition and its Treatment.

BY EMILE BRUNOR, M. D., NEW YORK.

Tuberculosis in its various forms has a very distinctive, inhibitory effect on nutrition. It causes a series of secondary symptoms which are sometimes harder to overcome than the original lesion.

We are told that any case of tuberculosis will get well if given plenty of food, fresh air and rest. Fresh air and rest are easy enough to obtain. but when we try to overfeed the patient we are confronted with a very difficult problem. The digestive organs are so impaired from this wasting disease that even a modicum of food cannot be assimilated.

Mal-nutrition may be due to several causes, as the profound toxemia caused by the bacillary infection, nervousness due to worry, reflex sexual neuroses, anemia, hepatic, renal or intestinal insufficiency. Sometimes we have one or more of the above symptoms to deal with, sometimes the disease has progressed to such an extent that all of these symptoms or causes are embodied in one case.

Cases of glandular tuberculosis are particularly amenable to surgical treatment provided the glands are superficial and not adherent to any vital structures. Sometimes the glandular or closed tuberculosis, if we may so call it, has involved the mesenteric glands and the lymphatics surrounding the abdominal or pelvic viscera. In those cases surgical treatment is not to be thought of inasmuch as it converts a latent or chronic tubercular case into an acute one. I wish to report a case of glandular tuberculosis involving the lymphatics throughout the gastro-intestinal tract. It is one which was referred to me for surgical treatment as a last resort.

G. S., age 17, born in America, of American parents. Has been suffering for the past four years from progressive debility. He was treated by various physicians from time to time for gastritis, constipation and diarrhea. Finally he was advised to go to Liberty. While there he suffered with violent diarrhea and indigestion. After a few months' stay his parents were convinced that his condition was aggravated rather than benefited by the climate. He was brought back to New York and he began to go down hill at a rapid pace.

When admitted in my hospital, in January, 1913, his weight was 60 pounds. In June, 1912, he weighed 115 pounds, showing a loss of 55 pounds in seven months. The physical examination showed no pulmonary involvement. The heart was dilated, pulse 140, urine had a high specific gravity, loaded with urates, uric acid and phosphates. The temperature was sub-normal, 95° F. His skin was drawn so tight over his bones that it would crack over the joints. The skin over the face was tightly drawn, so much so that his mouth was always open. The appearance of the body reminded one of the famine sufferers of India. He could not retain or assimilate any food. The smallest quantity of solid food caused a great deal of distress, nausea and gas. The first week after admittance to the hospital he was placed on a milk diet and, owing to the difficulty of digestion, the milk was diluted with vichy and given at frequent intervals, and in spite of that we had a great deal of difficulty in making him retain any.

Then, to make sure as to the etiology of the mal-nutrition, I gave him an injection of tuberculin, which caused the usual reaction, chill, temperature and sweat. Simultaneously with the tubercular test I used the Moro Percutaneous test and after three days it was mildly positive. It convinced me that this was a case of intestinal tuberculosis. There were mitral murmurs. The aorta gave a blowing sound which is typical of profound anemia.

By palpation one could feel hard mesenteric glands under the skin, a few lymphatics which were hard and enlarged and the skin was dry and very pale; the eyes had that pearly iridescent look found in anemia; the conjunctiva, the lips and gums were pale, the matrix of the finger nails was bluish and the palmar surface of the hands was cold and clammy.

After the first week we were able to use a couple of quarts of milk a day and an emulsion of bone marrow, eggs and malt extract. Daily enemas of soapsuds and saline solution were used to cleanse the bowels. A dose of castor oil was given about once a week, and the weight of the patient began to increase at the rate of five pounds a week.

I also gave him five intravenous injections of an isotonic solution, which contained: acid salicylic, mercury albuminate, iron, sodium chloride, calcium carbonate and phenols.

The injections were given every other week with great difficulty on account of the small lumen of the veins. However, the temperature went to normal, the cold clammy sweat gave way to a warm glow, the appetite improved and the increase in weight from that time on was at the rate of about a pound a day.

When the patient reached 100 pounds, this increase in weight was reduced to only two pounds a week. The emulsion of bone marrow, eggs and malt was discontinued after a month, as the patient's stomach rebelled against the monotony of the tonic, so we used another preparation composed of the gluten of wheat and malt.*

To overcome the deficient circulation, we gave him hot baths every night and a rub down with cocoanut oil to make the skin more supple. In addition, he then received a highly nutritious diet and occasionally an eggnog. At the end of the second month, when the patient weighed 120 pounds, I took him out for a walk to test his strength, and he walked five miles before feeling any

*Malt-Clidine.

Cuts reproduced by courtesy of Menbey & James, 168 Duane St., N. [illegible]

fatigue. I then weighed him and found he had not lost any appreciable amount. This encouraged me to provide him with a reasonable amount of physical exercise, and when he left the hospital three months after admission he weighed 130 pounds.

He went to the country for the summer, where he indulged in all kinds of strenuous exercises and sports, and his weight upon his return was 140 pounds. He is in perfect health, eats, sleeps and works as though he never had a sick day in his life.

Last month he contracted a little cold with a severe cough, and I watched him closely to see if there were any possibility of pulmonary involvement, but the cold disappeared and the only difference is that he has returned to his former weight of 130 pounds.

When his picture was taken upon admittance it was with difficulty he could stand up for the five minutes necessary to take his photgraph. Three months later the photograph speaks for itself.—*The Medical Times.*

---

### Indiana Child Creed.

Every child has the inalienable right to be born free from disease, free from deformity and with pure blood in its veins and arteries.

Every child has the inalienable right to be loved; to have its individuality respected; to be trained wisely in mind, body and soul; to be protected from disease, from evil influences and evil persons; and to have a fair chance in life. In a word, to be brought up in the fear and admonition of the Lord.

That State is delinquent which does not ceaselessly strive to secure these inalienable rights to its children.

## Figures and Tables

**Figure f1:**
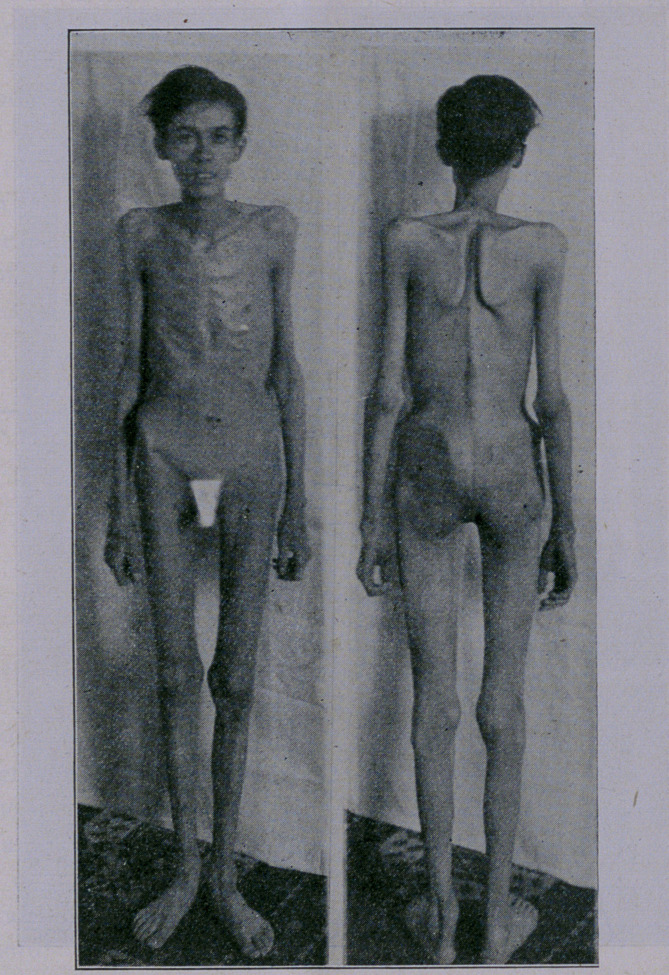


**Figure f2:**